# Dr. Roland G. Curtin

**Published:** 1913-05

**Authors:** 


Dr. Roland G. Curtin of Philadelphia, University of Pennsylvania, 1866, died March 14, aged 74. He was Naval Store

Keeper in Philadelphia during the Civil War and was Assistant Medical Director of the Centennial Exposition of 1876. He was well known as a clinician, many of our readers having had the privilege, which we enjoyed, of attending his clinics at the Blockley Hospital.

